# Tumor Phosphatidylinositol-3-Kinase Signaling and Development of Metastatic Disease in Locally Advanced Rectal Cancer

**DOI:** 10.1371/journal.pone.0050806

**Published:** 2012-11-30

**Authors:** Anne Hansen Ree, Annette Torgunrud Kristensen, Marie Grøn Saelen, Rik de Wijn, Hege Edvardsen, Jovana Jovanovic, Torveig Weum Abrahamsen, Svein Dueland, Kjersti Flatmark

**Affiliations:** 1 Department of Oncology, Akershus University Hospital, Lørenskog, Norway; 2 Institute of Clinical Medicine, University of Oslo, Oslo, Norway; 3 Department of Tumor Biology, Oslo University Hospital–Radium Hospital, Oslo, Norway; 4 PamGene International B.V., ‘s-Hertogenbosch, The Netherlands; 5 Department of Genetics, Oslo University Hospital–Radium Hospital, Oslo, Norway; 6 Department of Clinical Molecular Biology, Akershus University Hospital, Lørenskog, Norway; 7 Department of Oncology, Oslo University Hospital, Oslo, Norway; 8 Department of Gastroenterological Surgery, Oslo University Hospital–Radium Hospital, Oslo, Norway; University of South Alabama, United States of America

## Abstract

**Background:**

Recognizing EGFR as key orchestrator of the metastatic process in colorectal cancer, but also the substantial heterogeneity of responses to anti-EGFR therapy, we examined the pattern of composite tumor kinase activities governed by EGFR-mediated signaling that might be implicated in development of metastatic disease.

**Patients and Methods:**

Point mutations in *KRAS*, *BRAF*, and *PIK3CA* and *ERBB2* amplification were determined in primary tumors from 63 patients with locally advanced rectal cancer scheduled for radical treatment. Using peptide arrays with tyrosine kinase substrates, *ex vivo* phosphopeptide profiles were generated from the same baseline tumor samples and correlated to metastasis-free survival.

**Results:**

Unsupervised clustering analysis of the resulting phosphorylation of 102 array substrates defined two tumor classes, both consisting of cases with and without *KRAS/BRAF* mutations. The smaller cluster group of patients, with tumors generating high *ex vivo* phosphorylation of phosphatidylinositol-3-kinase-related substrates, had a particularly aggressive disease course, with almost a half of patients developing metastatic disease within one year of follow-up.

**Conclusion:**

High phosphatidylinositol-3-kinase-mediated signaling activity of the primary tumor, rather than *KRAS/BRAF* mutation status, was identified as a hallmark of poor metastasis-free survival in patients with locally advanced rectal cancer undergoing radical treatment of the pelvic cavity.

## Introduction

The multitude of more than 500 protein kinases, the kinome, represents a substantial part of the human genome, and receptor tyrosine kinases are key mediators in signaling cascades regulating central biological processes of malignancy, such as proliferation, angiogenesis, and metastasis [1], [2]. In order to optimize and individualize therapeutic efficacy of kinase inhibiting agents for metastatic disease control, it seems rational to exploit the specific pattern of tumor kinase activity as functional biomarker of actionable targets.

In locally advanced rectal cancer (LARC), randomized studies have highlighted the central role of chemoradiotherapy (CRT) in conjunction with surgical resection to eradicate tumor within the pelvic cavity and improve long-term outcome [3]. However, even with successful local treatment, a substantial number of patients will develop metastatic disease as result of early, undetected systemic dissemination of tumor cells. Within this frame of reference, our prospective non-randomized study comprising LARC patients given CRT followed by radical surgery and no further treatment offers a unique opportunity to explore the regulatory role of specific kinase signaling pathways in tumor proliferation, angiogenesis, and metastasis in a defined clinical context. In this study, using peptide arrays with tyrosine kinase substrates [4]–[6] to analyze the patients’ tumors at the time of diagnosis, we have found that patients with poor CRT response had significantly elevated tumor kinase activity, representing signaling mediated by VEGFR, EGFR, and phosphatidylinositol-3-kinase (PI3K)/AKT, compared to good-responding patients [7]. Moreover, we have reported that tumor angiogenic signatures comprising PDGFR, VEGFR, and EPOR were associated with microscopic dissemination of tumor cells in bone marrow at the time of diagnosis, which secondly was correlated with heightened risk of developing metastatic disease following the course of radical treatment of the pelvic cavity [8].

In metastatic colorectal cancer, monoclonal antibodies directed against EGFR, currently cetuximab and panitumumab, have been implemented in clinical practice for the last eight years. For the optimum selection of eligible patients, initial molecular data established mutations of genes encoding effector proteins downstream of EGFR in the tumor signaling cascade, primarily mutations in codon 12 or 13 of *KRAS*, as predictor of intrinsic therapeutic resistance to anti-EGFR monoclonal antibodies [9]. Moreover, mutations in genes encoding other mediators, primarily *BRAF* p.V600E but also *PIK3CA* mutations, are associated with resistance [9], while tumors harboring *KRAS* p.G13D may respond [10], [11]. It was recently suggested that amplification of *ERBB2* comprises another resistance mechanism [12], [13], and that acquired resistance is conferred by mutation of *EGFR* itself [14] or results from expansion of tumor subclones with mutated or amplified *KRAS* [15], [16]. In addition to signaling through the pathway of *KRAS/BRAF*-encoded effectors, EGFR in parallel activates PI3K-mediated signaling through a multistep interplay of messenger molecules with effectors [17], [18]. Despite all of the above knowledge, however, objective response to anti-EGFR antibody therapy in metastatic colorectal cancer is obtained, at best, in no more than half of patients without tumor aberrations in the EGFR signaling network that are currently tested in routine practice [9], [19], [20].

Hypothesizing that kinase signaling activity conducted by EGFR may reflect mutation status of genes encoding effector proteins from any component of the molecular network, we compared the previously achieved *ex vivo* tumor phosphopeptide profiles from the LARC study patients [7], [8] with tumor mutations within *KRAS* exon 2, *BRAF* exon 15, and *PIK3CA* exons 9 and 20, and amplification of *ERBB2*. Conceptually, tumor kinase activity signatures comprising all interacting signaling pathways of relevance might be developed into functional biomarkers of actionable therapy targets for metastatic disease control. The investigations in this study defined a subgroup of LARC patients, following the resection of primary tumors with high activity of the PI3K signaling pathway, with particularly poor metastasis-free survival. This finding suggests that high tumor PI3K-mediated signaling activity is a biomarker of risk assessment and treatment stratification.

## Materials and Methods

### Ethics Statement

The phase II, non-randomized study protocol (ClinicalTrials ID NCT00278694) was approved by the Institutional Review Board and the Regional Committee for Medical and Health Research Ethics of South-East Norway, and is in accordance with the Helsinki Declaration. Written informed consent was required for participation.

### Patients and Procedures

The patient population reported here was enrolled between October 2005 and May 2008. Patient eligibility criteria, evaluation procedures, study treatment, and review procedures of follow-up have been described in detail previously [7]. Following neoadjuvant fluoropyrimidine−/oxaliplatin-based CRT and subsequent surgery, the resected primary tumor specimens were histologically evaluated for treatment response according to standard criteria (histopathologic staging; ypTN) and histomorphologic tumor regression grade (TRG). Briefly, the latter was graded within one of five TRG categories, spanning from the absence of residual tumor cells in the resected specimen (TRG1) to the lack of morphologic signs of treatment response (TRG5) [21]. Follow-up data was obtained from the clinical database and censored on December 31, 2011. Valid observations of the presence or absence of distant metastases required designated radiologic examination. Four patients with synchronous resectable liver metastases were excluded from analysis of metastasis-free survival.

### Tumor Samples

At the time of diagnosis, baseline study-specific primary tumor biopsies were obtained from 79 patients with locally advanced rectal cancer under heavy sedation, snap-frozen in liquid nitrogen, and stored at –80°C, as reported previously [7]. Of the included patients, 16 patients were excluded from the present study, as 12 patients had tumor biopsy specimens in which kinase activity profiling had not been performed because the patients were either ineligible after study registration (*n* = 4), had withdrawn consent (*n* = 1), had unexpectedly died during the preoperative treatment (*n* = 1), had developed metastatic disease progression during preoperative treatment that precluded definitive surgery (*n* = 1), had tumor cell content less than 20% within the biopsy specimen (*n* = 3), or had a biopsy specimen in which kinase activity analysis was missing of unknown reasons (*n* = 2), and four additional patients did not have tumor DNA isolated because no biopsy material remained for the purpose. Thus, tumor kinase activity profiles based on previous array phosphosubstrate data were successfully identified for 63 patients that had their tumor *KRAS/BRAF/PIK3CA/ERBB2* mutation status determined, and this study population was present within the current analyses.

### Tumor Gene Mutation Analyses

*KRAS*, *BRAF*, and *PIK3CA* target sequences were amplified by polymerase chain reaction, and base substitutions were detected by denaturant, cycling temperature capillary electrophoresis [22], [23], according to Table S1. *ERBB2* amplification was analyzed using the TaqMan® Copy Number Assay (Applied Biosystems, Oslo, Norway) protocol [24] and calibrated relative to each individual patient’s corresponding DNA isolated from peripheral blood mononuclear cells. Tumor DNA samples with relative quantification values higher than 5 were considered amplified to ensure scoring high-grade focal *ERBB2* amplification only, omitting low-grade polysomy of chromosome 17.

### Tumor Kinase Activity Profiling

Preparation of tumor sample lysates and multiplex analysis of tumor kinase activity using peptide arrays with tyrosine kinase substrates (Tyrosine Kinase PamChip96 Array; PamGene International B.V.,‘s-Hertogenbosch, The Netherlands) have been described in detail previously [7]. The average tumor cell content in the biopsy specimens was 46%, and no difference was found between tumors with wild-type and mutated *KRAS/BRAF* (*P*>0.67; two-sample *t*-test). Four technical replicates were analyzed from each patient sample to generate *ex vivo* phosphosubstrate profiles.

### Adaptation of Array Data

Data visualization and processing of previously achieved array data (ArrayExpress accession number E-TABM-913), as reported previously [7], were performed using BioNavigator version 5.10.70 (PamGene International B.V.). The tumors were divided into two groups; wild-type and mutated *KRAS/BRAF* status (36 and 27 samples, respectively). The data on array peptide phosphorylation, following conversion from array signal intensities, was log-transformed after handling a small number of negative data points by subtracting the 1% percentile of the total data set and subsequently setting all remaining data points with value less than 1 to the value 1. This adaptation approach was chosen to balance the number of data points that was set to the value of 1 and the extent of collective upward shift of the whole data set. Correction of plate-to-plate variation was achieved by normalizing substrate signal intensity to the mean signal intensity of all wild-type tumors in the respective plates by the following formula: N_psm_ = log_2_(S_psm_) – log_2_(G_pm_), where N_psm_ is the normalized signal for substrate p of sample s on plate m, S_psm_ is the corresponding non-normalized signal, and G_pm_ is the average signal from wild-type tumors of substrate p on plate. Phosphosubstrates with a sample-average signal less than 10 were excluded, leaving 102 peptides for further analysis (Table S2).

### Statistical Analysis

Based on the signal values of these resultant 102 array phosphopeptides, unsupervised analysis was performed applying principal component analysis and *k*-means clustering, with 10 Monte Carlo repetitions, using standard functions provided in the Matlab Statistics Toolbox (Matlab R2010A; Mathworks, Natick, MA, USA). Binary supervised classification analysis was performed using partial-least squares discriminant analysis in Matlab R2010A, essentially as described previously [7]. Performance of class partition with respect to tumor mutation status was evaluated by 20-fold cross validation. Distribution of parameters between patients with tumors harboring differential molecular features was compared using Fisher’s exact test for categorical data and two-sample *t*-test for continuous variables. Log-rank test was applied to calculate any difference in metastasis-free survival between patient subgroups. The data analysis was performed using SPSS Predictive Analytics Software (SPSS Inc., Chicago, IL, USA). *P*-values less than 0.05 were considered statistically significant.

## Results

### Tumor Mutations

Point mutations in *KRAS*, *BRAF*, and *PIK3CA* and amplification of *ERBB2* were detected in 35%, 6.3%, 9.5%, and 3.2% of the primary tumors from 63 LARC cases, respectively (Table 1); in the majority, a single gene aberration was found. Four tumors harbored *BRAF* mutation, either p.D594G or p.V600E, as a solitary aberration. In six tumors, *PIK3CA* mutations were found; in two of the cases, *KRAS* was also mutated. Only two samples, both without other detected mutations, showed amplified *ERBB2* (by virtue of higher than 5-fold tumor *ERBB2* level relative to the level in the patient’s corresponding normal DNA). No differences were observed between patients harboring *KRAS/BRAF* wild-type and mutated tumors regarding radiologic TNM stage at diagnosis, histopathologic ypTN stage or histomorphologic TRG score of the surgical specimens following CRT, development of metastatic disease at median follow-up of 53 months (range 7–70), or age (Table S3).

**Table 1:** Frequencies of tumor *KRAS*, *BRAF*, and *PIK3CA* point mutations and *ERBB2* amplification in 63 patients with locally advanced rectal cancer.

Mutations		*n* (%)
*KRAS* exon 2		22 (35)
	p.G12D	8 (13)
	p.G12V	6 (9.5)
	p.G13D	3 (4.8)
	p.G12C	2 (3.2)
	p.G12S	1 (1.6)
	p.G13S	1 (1.6)
	unspecified	1 (1.6)
*BRAF* exon 15		4 (6.3)
	p.D594G	2 (3.2)
	p.V600E	2 (3.2)
*PIK3CA*		6 (9.5)
	exon 9	5 (7.9)
	exon 20	2 (3.2)
*ERBB2*		2 (3.2)

### Tumor Kinase Activity Profiles

*Ex vivo* tumor kinase activity profiles were derived from 102 array substrates that had signal intensities above the defined threshold (Table S2), with relative phosphorylation levels varying within a log_2_ range of –1.0 to 1.0. Based on the generated phosphosubstrate profiles, a binary class partition model discriminated correctly between tumor *KRAS/BRAF* wild-type and mutation status in 67% of cases. No improvement in precision of class partition was achieved on inclusion of either *PIK3CA* or *ERBB2* aberrations as additional layers of information to the group of tumor samples with gene mutations.

**Figure 1:**
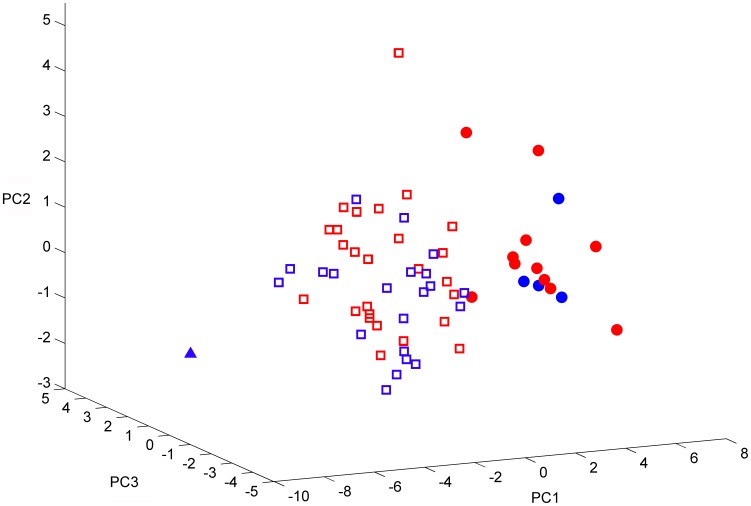
Tumor kinase activity cluster groups in patients with locally advanced rectal cancer. Unsupervised clustering analysis of kinase substrate phosphorylation levels generated by tumors from 63 patients. Distribution of the individual samples of *KRAS/BRAF* wild-type (red) and mutated (blue) tumors is visualized using the scores of the first three components in a principal component analysis (PC1–3) of the range of phosphorylation levels of 102 *ex vivo* kinase substrates. *k*-means clustering was used to obtain two groups of tumor samples, indicated by open squares (Cluster-Group 1) and closed circles (Cluster-Group 2), respectively. The closed triangle represents a single outlier to the distribution of samples along PC1, as elaborated in Results.

Figure 1 shows the score plot resulting from principal component analysis of the data set of 102 phosphopeptide substrates; each spot represents one of the 63 samples in a three-dimensional principal component space. On inspection of the score plot, a single tumor (closed triangle) was observed as a clear outlier to the distribution of samples along the first principal component, and furthermore, the remaining samples seemed to separate into two groups, both consisting of a relatively balanced number of *KRAS/BRAF* wild-type and mutated tumors. Subsequently, using the scores of the three principal components as input and excluding the outlier tumor from further analysis, *k*-means clustering was applied in order to obtain two distinct groups of samples, thereby assigning any borderline cases into either of the two. Resulting from this procedure, 15 of 62 samples (24%), of which 11 (69% of the 15) were *KRAS/BRAF* wild-type cases, clustered in the smaller group (Cluster-Group 2; closed circles). Although the larger cluster of 47 samples (Cluster-Group 1; open squares) consisted of 26 (55%) *KRAS/BRAF* wild-type tumors, *KRAS/BRAF* wild-type and mutated cases were equally distributed within the two tumor clusters (*P* = 0.17).

**Figure 2:**
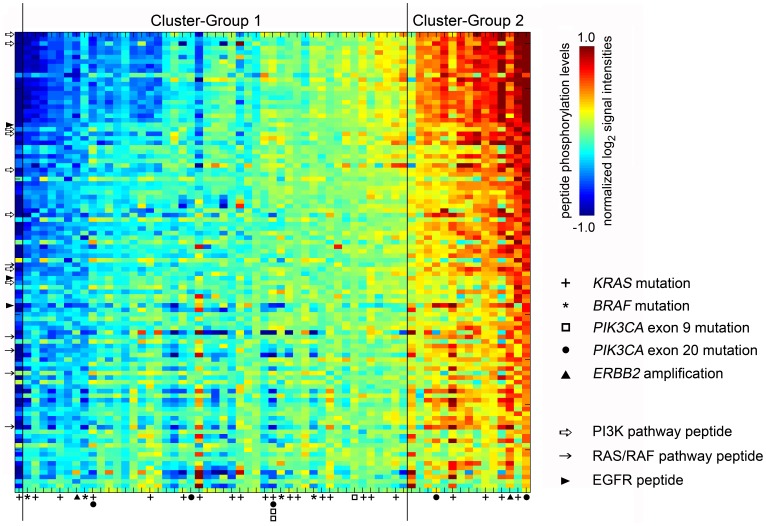
Tumor *ex vivo* phosphorylation profiles from patients with locally advanced rectal cancer. An imaginary line was drawn between the determined centroid of each of the patient Cluster-Group 1 and Cluster-Group 2 (depicted in Figure 1), and the 63 tumor samples (horizontal axis; marked for gene mutations as specified) and 102 phosphosubstrates (vertical axis) were sorted along this line according to projection and weight in signal difference, respectively. Red corresponds to higher and blue to lower substrate phosphorylation levels. Arrows denote array peptides representing factors of EGFR-directed signaling pathways, as indicated, and the identity of each peptide substrate, in order from top to bottom of the figure, is given in Table 2. In this analysis, the single outlier to the distribution of samples, as elaborated in Results, sorted left of Cluster-Group 1 in the heat-map.

In Figure 2, using the resulting groups from the unsupervised clustering analysis, tumor samples (horizontal axis) and peptides (vertical axis) were sorted along a line connecting the two cluster centroids according to their projection and weight in signal change, respectively, illustrating that Cluster-Group 2 tumors generated higher *ex vivo* substrate phosphorylation levels than the samples of Cluster-Group 1 for all of the 102 peptides. The order of the peptide substrates with respect to how their difference in phosphorylation levels across the tumor samples distinguished between the two cluster groups, *i.e.,* the discriminating tumor kinase activity profile, is listed in Table 2. Interestingly, differences in phosphorylation of substrates related to PI3K-dependent factors (one PIK3R1, three CTTN1, one PLCG1, two PDPK1, and one RASA1 peptides) contributed more strongly to the discrimination of cluster groups, as they essentially were found in the upper half of the list, than phosphosubstrates related to signaling mediated by the *KRAS/BRAF*-encoded effector pathway (one RAF1 and four MAPK isoforms peptides). One of the three EGFR array peptides was found among phosphosubstrates strongly distinguishing between the two cluster groups.

**Table 2:** Order of the 102 array phosphosubstrates, listed from highest to lowest difference in phosphorylation level (top to bottom in Figure 2) between Cluster-Group 1 and Cluster-Group 2 tumors from 62 patients with locally advanced rectal cancer.

Substrate identity a	Peptide sequence	Tyrosine position b	Common name a
**PIK3R1**	NENTEDQYSLVED	[607]	**Phosphatidylinositol 3-kinase regulatory alpha subunit**
FES	REEADGVYAASGG	[713]	Proto-oncogene tyrosine-protein kinase Fes/Fps
**CTTN1**	EYEPETVYEVAGA	[477, 483]	**Src substrate protein p85**
CDK2	EKIGEGTYGVVYK	[15], [19]	Cell division protein kinase 2
PXN	VGEEEHVYSFPNK	[118]	Paxillin
VEGFR2(KDR)	EEAPEDLYKDFLT	[996]	Vascular endothelial growth factor receptor 2
EPHA2	EDDPEATYTTSGG	[772]	Ephrin type-A receptor 2
EPHA1	LDDFDGTYETQGG	[781]	Ephrin type-A receptor 1
PXN	FLSEETPYSYPTG	[31], [33]	Paxillin
PECAM1	KKDTETVYSEVRK	[713]	Platelet endothelial cell adhesion molecule
EPHA7	TYIDPETYEDPNR	[608, 614]	Ephrin type-A receptor 7
CD247	KDKMAEAYSEIGM	[123]	T-cell surface glycoprotein CD3 zeta chain
FRK	KVDNEDIYESRHE	[387]	Tyrosine-protein kinase FRK
EPHB1	DDTSDPTYTSSLG	[778]	Ephrin type-B receptor 1
EPOR	ASAASFEYTILDP	[426]	Erythropoietin receptor
RET	TPSDSLIYDDGLS	[1029]	Proto-oncogene tyrosine-protein kinase receptor ret
EPOR	SEHAQDTYLVLDK	[368]	Erythropoietin receptor
PDGFRB	VSSDGHEYIYVDP	[579, 581]	Beta platelet-derived growth factor receptor
LAT	EEGAPDYENLQEL	[255]	Linker for activation of T cells
FER	RQEDGGVYSSSGL	[714]	Proto-oncogene tyrosine-protein kinase FER
***EGFR***	GSVQNPVYHNQPL	[1110]	***Epidermal growth factor receptor***
**PLCG1**	IGTAEPDYGALYE	[771, 775]	**1-phosphatidylinositol-4,5-bisphosphate phosphodiesterase gamma 1**
**PDPK1**	ARTTSQLYDAVPI	[9]	**3-phosphoinositide dependent protein kinase 1**
PDGFRB	PNEGDNDYIIPLPDP	[1021]	Beta platelet-derived growth factor receptor
CBL	EGEEDTEYMTPSS	[700]	CBL E3 ubiquitin protein ligase
LAT	MESIDDYVNVPES	[200]	Linker for activation of T cells
PDGFRB	SSNYMAPYDNYVP	[771, 775, 778]	Beta platelet-derived growth factor receptor
PDGFRB	LDTSSVLYTAVQP	[1009]	Beta platelet-derived growth factor receptor
TNNT1	SDTEEQEYEEEQP	[9]	Slow skeletal muscle troponinT
KRT6E	GAGFGSRSLYGLG	[62]	Keratin, type II cytoskeletal 6E
**RASA1**	TVDGKEIYNTIRR	[460]	**Ras GTPase-activating protein 1**
PDGFRB	YMAPYDNYVPSAP	[771, 775, 778]	Beta platelet-derived growth factor receptor
ANXA2	HSTPPSAYGSVKA	[24]	Annexin A2
PTK2B	RYIEDEDYYKASV	[573, 579, 580]	Protein tyrosine kinase 2 beta
PDGFRB	RPPSAELYSNALP	[716]	Beta platelet-derived growth factor receptor
JAK1	AIETDKEYYTVKD	[1022, 1023]	Tyrosine-protein kinase JAK1
ZAP70	ALGADDSYYTARS	[492, 493]	Tyrosine-protein kinase ZAP-70
DDR1	LLLSNPAYRLLLA	[513]	Epithelial discoidin domain receptor 1
CTNNB1	VADIDGQYAMTRA	[86]	Beta-catenin
JAK2	VRREVGDYGQLHETE	[570]	Tyrosine-protein kinase JAK2
**CTTN1**	YQAEENTYDEYEN	[492, 499, 502]	**Src substrate protein p85**
FGFR2	TLTTNEEYLDLSQ	[769]	Fibroblast growth factor receptor 2
MET	RDMYDKEYYSVHN	[1230, 1234, 1235]	Hepatocyte growth factor receptor
ART-004	EAIYAAPFAKKK	[4]	Artificial peptide sequence
NTRK2	GMSRDVYSTDYYR	[702, 706, 707]	BDNF/NT-3 growth factors receptor
VEGFR1 (FLT1)	DYNSVVLYSTPPI	[1327, 1333]	Vascular endothelial growth factor receptor 1
ANXA1	IENEEQEYVQTVK	[21]	Annexin A1
MST1R	SALLGDHYVQLPA	[1353]	Macrophage-stimulating protein receptor
LCK	RLIEDNEYTAREG	[394]	Proto-oncogene tyrosine-protein kinase LCK
VEGFR2(KDR)	AQQDGKDYIVLPI	[1175]	Vascular endothelial growth factor receptor 2
ERBB2	LDIDETEYHADGG	[877]	Receptor tyrosine-protein kinase erbB-2
*MAPK7*	AEHQYFMTEYVAT	[215, 220]	*Mitogen-activated protein kinase 7*
**PDPK1**	DEDCYGNYDNLLS	[373, 376]	**3-phosphoinositide dependent protein kinase 1**
PRRX2	WTASSPYSTVPPY	[208, 214]	Paired mesoderm homeobox protein 2
***EGFR***	ISLDNPDYQQDFF	[1172]	***Epidermal growth factor receptor***
**CTTN1**	VSQREAEYEPETV	[477]	**Src substrate protein p85**
MST1R	YVQLPATYMNLGP	[1353, 1360]	Macrophage-stimulating protein receptor
EPB41	LDGENIYIRHSNL	[660]	Protein 4.1
CHRND	YISKAEEYFLLKS	[383, 390]	Acetylcholine receptor protein, delta subunit
ERBB2	PTAENPEYLGLDV	[1248]	Receptor tyrosine-protein kinase erbB-2
***EGFR***	STAENAEYLRVAP	[1197]	***Epidermal growth factor receptor***
CALM1	KDGNGYISAAELR	[100]	Calmodulin
FGFR1	TSNQEYLDLSMPL	[766]	Basic fibroblast growth factor receptor 1
DCX	GIVYAVSSDRFRS	[112]	Neuronal migration protein doublecortin
FGFR3	TVTSTDEYLDLSA	[760]	Fibroblast growth factor receptor 3
VEGFR1(FLT1)	ATSMFDDYQGDSS	[1242]	Vascular endothelial growth factor receptor 1
TEC	RYFLDDQYTSSSG	[513, 519]	Tyrosine-protein kinase Tec
*RAF1*	PRGQRDSSYYWEI	[340, 341]	*RAF proto-oncogene serine/threonine-protein kinase*
PGR	EQRMKESSFYSLC	[795]	Progesterone receptor (PR)
BCKDHA	DDSSAYRSVDEVN	[345]	2-oxoisovalerate dehydrogenase alpha subunit, mitochondrial
*MAPK10*	TSFMMTPYVVTRY	[223]	*Mitogen-activated protein kinase 10*
DYRK1A	CQLGQRIYQYIQS	[319, 321]	Dual-specificity tyrosine-phosphorylation regulated kinase 1A
ERBB4	IVAENPEYLSEFS	[1284]	Receptor tyrosine-protein kinase erbB-4
VEGFR2(KDR)	DIYKDPDYVRKGD	[1054, 1059]	Vascular endothelial growth factor receptor 2
RB1	IYISPLKSPYKIS	[805, 813]	Retinoblastoma-associated protein
*MAPK1*	HTGFLTEYVATRW	[187]	*Mitogen-activated protein kinase 1*
INSR	YASSNPEYLSASD	[992, 999]	Insulin receptor
PTK2	RYMEDSTYYKASK	[570, 576, 577]	Focal adhesion kinase 1
EPHA4	LNQGVRTYVDPFT	[596]	Ephrin type-A receptor 4
EPHB4	IGHGTKVYIDPFT	[590]	Ephrin type-B receptor 4
VCL	KSFLDSGYRILGA	[822]	Vinculin
SYK	ALRADENYYKAQT	[525, 526]	Spleen tyrosine kinase
VEGFR1(FLT1)	DFGLARDIYKNPD	[1048]	Vascular endothelial growth factor receptor 1
C1R	TEASGYISSLEYP	[204, 210]	Complement C1r subcomponent
MBP	ARTAHYGSLPQKS	[203]	Myelin basic protein
PPP2CB	EPHVTRRTPDYFL	[307]	Serine/threonine protein phosphatase 2A, catalytic subunit, beta isoform
VEGFR2(KDR)	DFGLARDIYKDPD	[1063]	Vascular endothelial growth factor receptor 2
*MAPK12*	ADSEMTGYVVTRW	[185]	*Mitogen-activated protein kinase 12*
SLC34A1	AKALGKRTAKYRW	[511]	Renal sodium-dependent phosphate transport protein 2
ZBTB16	LRTHNGASPYQCT	[630]	Zinc finger and BTB domain containing protein 16
CDK7	GLAKSFGSPNRAY	[169]	Cell division protein kinase 7
VEGFR3(FLT4)	DIYKDPDYVRKGS	[1063, 1068]	Vascular endothelial growth factor receptor 3
TYRO3	KIYSGDYYRQGCA	[681, 685, 686]	Tyrosine-protein kinase receptor TYRO3
VEGFR2(KDR)	RFRQGKDYVGAIP	[951]	Vascular endothelial growth factor receptor 2
NCF1	QRSRKRLSQDAYR	[324]	Neutrophil cytosol factor 1
MBP	FGYGGRASDYKSA	[261, 268]	Myelin basic protein
PTPN11	SKRKGHEYTNIKY	[546, 551]	Tyrosine-protein phosphatase, non-receptor type 11
NTRK1	HIIENPQYFSDAC	[496]	High affinity nerve growth factor receptor
MBP	GRASDYKSAHKGF	[268]	Myelin basic protein
ENPEP	EREGSKRYCIQTK	[12]	Glutamyl aminopeptidase
VEGFR1(FLT1)	KNPDYVRKGDTRL	[1053]	Vascular endothelial growth factor receptor 1
INSR	SLGFKRSYEEHIP	[1355]	Insulin receptor

Peptides representing phosphatidylinositol-3-kinase-dependent factors are indicated in bold, whereas peptides related to signaling mediated by the *KRAS/BRAF*-encoded effector pathway are italicized. The EGFR peptides are highlighted in bold and italics.

a Retrieved from UniProtKB/SwissProt (http://au.expasy.org/sprot).

b Position(s) of the tyrosine phosphorylation site(s) within the protein.

**Figure 3:**
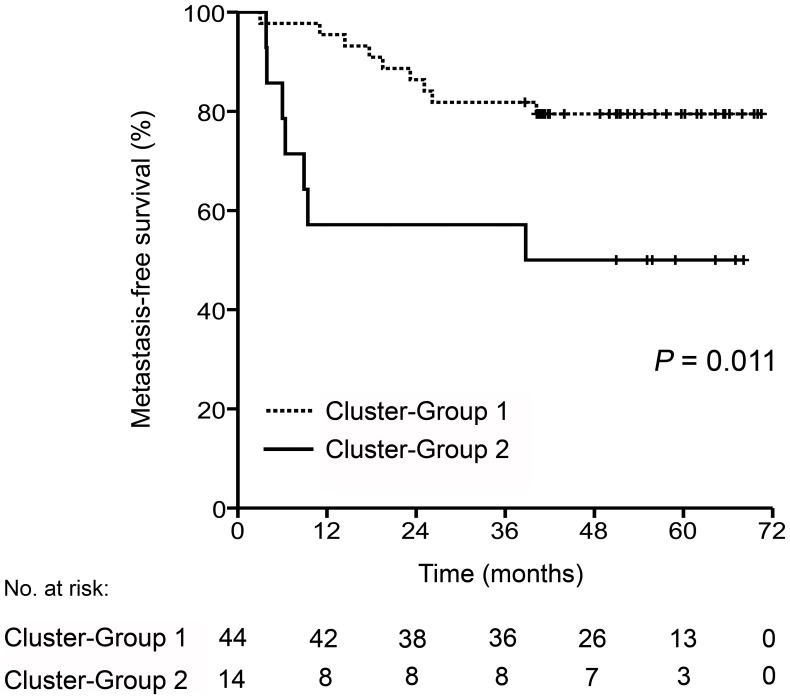
Metastasis-free survival in locally advanced rectal cancer. This outcome parameter was analyzed for 58 patients as function of low (Cluster-Group 1) or high (Cluster-Group 2) *ex vivo* substrate phosphorylation activity of the primary tumor.

Table 3 summarizes tumor and treatment characteristics of the 62 patients included in either Cluster-Group 1 or Cluster-Group 2. Again, no differences were observed between the cluster groups regarding TNM stage, ypTN stage, or TRG score. Metastasis-free survival was assessed for 58 patients, as the four patients with synchronous liver metastases were omitted from this analysis, with Cluster-Group 2 demonstrating poorer metastasis-free survival than Cluster-Group 1 (*P* = 0.011; Figure 3). Of particular note, whilst a fifth of patients in Cluster-Group 1 developed metastatic disease over a follow-up period of 36 months, patients in Cluster-Group 2 seemed to have a much more aggressive disease, as almost a half had been diagnosed with metastases by less than one year of follow-up.

**Table 3:** Tumor and treatment characteristics of 62 patients with locally advanced rectal cancer.

		All patients in sample clusters (*n* = 62)	Patients in Cluster-Group 1 (*n* = 47)	Patients in Cluster-Group 2 (*n* = 15)
		*n* (%)	*n* (%)	*n* (%)
TNM				
	T2	4 (6.5%)	4 (8.5%)	0 (0%)
	T3	36 (58%)	29 (62%)	7 (47%)
	T4	22 (35%)	14 (30%)	8 (53%)
	N0	8 (13%)	8 (17%)	0 (0%)
	N1	9 (15%)	6 (13%)	3 (20%)
	N2	45 (73%)	33 (70%)	12 (80%)
	M0	57 (92%)	44 (94%)	13 (87%)
	M1	5 (8.1%)	3 (6.4%)	2 (13%)
ypTN				
	ypT0	13 (21%)	10 (21%)	3 (20%)
	ypT1	6 (10%)	3 (6.4%)	3 (20%)
	ypT2	16 (26%)	15 (32%)	1 (6.7%)
	ypT3	15 (24%)	12 (26%)	3 (20%)
	ypT4	12 (19%)	7 (15%)	5 (33%)
	ypN0	49 (79%)	38 (81%)	11 (73%)
	ypN1	10 (16%)	8 (17%)	2 (13%)
	ypN2	3 (4.8%)	1 (2.1%)	2 (13%)
TRG a				
	1–2	45 (73%)	35 (74%)	10 (67%)
	3	9 (15%)	8 (17%)	1 (6.7%)
	4–5	8 (13%)	4 (8.5%)	4 (27%)
Development of metastatic disease b		16 (26%)	9 (20%)	7 (50%)

a Tumor Regression Grade following chemoradiotherapy.

b Censored at a median period of 53 months (range 7–70), excluding four patients with synchronous resectable liver metastases.

**Figure 4:**
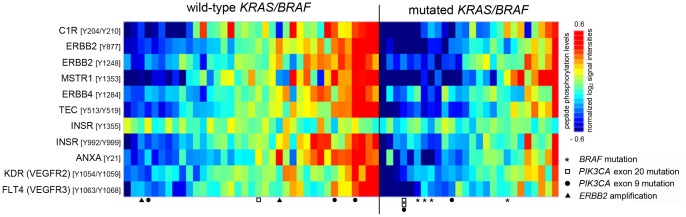
Tumor *ex vivo* phosphorylation profiles discriminating tumor *KRAS/BRAF* mutation status in locally advanced rectal cancer. The 63 tumor samples are ordered along the horizontal axis, annotated by wild-type or mutated *KRAS/BRAF* and marked for other gene mutations as specified, while the 11 discriminating kinase substrates (*P*-value range 0.0034–0.049 on comparison of *KRAS/BRAF* wild-type tumors as a whole group with the entire group of tumors harboring such mutations) are depicted along the vertical axis. For each peptide substrate, position(s) of the tyrosine phosphorylation site(s) within the protein is indicated. Red corresponds to higher and blue to lower substrate phosphorylation levels.

Separately, when comparing *KRAS/BRAF* wild-type tumors as a whole group with the entire group of tumors harboring such mutations, samples without mutations generated significantly higher phosphorylation of 11 of the 102 array peptides constituting the tyrosine kinase activity profiles (*P*-value range 0.0034–0.049). In Figure 4, the samples within each of these two tumor groups are organized horizontally in order from low to high phosphopeptide levels, to visualize the higher percentage of *KRAS/BRAF* wild-type tumors that produced higher than mean phosphorylation levels for the 11 substrates (18 of 36 samples) than tumors with mutated *KRAS/BRAF* performing correspondingly (6 of 27 samples; *P* = 0.036). A majority of the discriminating phosphosubstrates was deemed to represent signaling factors that are interconnected with the EGFR-conducted pathway. Within the entire 102-peptide panel, six peptides representing members of the EGFR family of receptor tyrosine kinases (three EGFR, two ERBB2, and one ERBB4) were identified (Table S2). Whilst none of the three EGFR array peptides was found among phosphosubstrates distinguishing tumor *KRAS/BRAF* mutation status at group level, all of the three peptides representing ERBB2 and ERBB4 were among the discriminating substrates.

## Discussion

The essential finding of this study was that high *ex vivo* phosphorylation of PI3K-related substrates by the primary tumor, rather than the *KRAS/BRAF* mutation status, may be a hallmark of poor metastasis-free survival in LARC patients after radical treatment of the pelvic cavity. This particular subgroup of study patients had aggressive disease development, with a substantial fraction of patients being diagnosed with metastatic disease within less than one year of follow-up.

Whilst contemporary multimodal treatment of LARC has led to significant improvement of local disease control, development of metastatic disease is still a major challenge [3]. Currently, no consensus exists to whether systemic therapy may reduce the risk of metastasis development in rectal cancer patients [25], which partly might be explained by the paucity of biomarkers for risk assessment and treatment stratification. In the patient cohort analyzed here, we have previously shown that the presence of disseminated tumor cells in bone marrow at the time of diagnosis correlated with development of overt metastatic disease [8]. The question of whether tumor *KRAS/BRAF* mutation status may be a reliable biomarker for the purpose of selecting high-risk patients to anti-EGFR therapy, however, remains elusive. Despite convincing evidence of efficacy in metastatic disease from wild-type *KRAS/BRAF* colorectal tumors [9], including the finding of a high percentage of resectability of liver metastases following cetuximab-based systemic therapy [26], the addition of cetuximab to standard chemotherapy in patients with wild-type *KRAS* colon cancer failed to meet the endpoint of prolonged disease-free survival in a recently concluded randomized trial in the adjuvant setting [27]. A similar study for resected rectal cancer has not been done.

In the present study, binary supervised classification analysis of the *ex vivo*-generated phosphopeptide profiles discriminated correctly between tumor *KRAS/BRAF* wild-type and mutated samples in two-thirds of cases. Because this particular data handling was performed to enable the detection of subtle differences between the two groups being compared, it did not fully compare with the outcome of the unsupervised analysis of the entire 102-phosphosubstrate panel. In the latter, two alternative phenotypic tumor populations appeared; a smaller one, comprising a fourth of the entire cohort, and a larger cluster of tumors, both consisting of samples with and without *KRAS/BRAF* mutations with similar distribution within the two tumor clusters. In metastatic colorectal cancer, objective response to anti-EGFR antibody therapy can be expected in a third of unselected patients, and conversely, tumor *KRAS* mutations may be found in almost a third of responders [19]. Our observation that *KRAS/BRAF* wild-type and mutated tumors had overlapping kinase activity profiles is consistent with the increasing recognition of tumor heterogeneity, reflected in disparate mutation status, as determinant of variable response to anti-EGFR antibody therapy.

Of notice, study patients demonstrating high tumor PI3K-mediated signaling activity, as all array substrates of this specific pathway (PIK3R1, CTTN1, PLCG1, PDPK1, and RASA1) were highly phosphorylated, had particularly poor metastasis-free survival after radical treatment of the pelvic cavity. The PI3K complex consists of a regulatory subunit, existing in several isoforms (PIK3R1 and CTTN1), and a catalytic subunit encoded by *PIK3CA*. On regulatory subunit phosphorylation by receptor tyrosine kinases or G-protein (RASA1)-coupled receptors, the catalytic subunit is enabled to generate phosphatidylinositol-3,4,5-trisphosphate, which activates 3-phosphoinositide-dependent protein kinase 1 (PDPK1) and subsequently AKT and the downstream mammalian target of rapamycin (mTOR). In addition, the 1-phosphatidylinositol-4,5-bisphosphate phosphodiesterase gamma-1 (PLCG1) is crucial for generation of activating second messenger molecules in the EGFR-directed, PIK3-mediated signaling pathway [17], [18]. In the context of our observations, even without valid evidence at present, it is tempting to speculate that LARC patients might be eligible for adjuvant systemic therapy based on this high-risk biological feature. Given the finding that the PI3K complex may be a key signaling network orchestrator of colorectal cancer metastasis, therapeutics targeting PI3K/AKT or the downstream mTOR complex [17] might be rational, and within this frame of reference, the *ex vivo* phosphosubstrate technology could show useful in developing the required biomarkers of signaling pathway druggability.

In the separate analysis comparing *ex vivo* phosphopeptide profiles generated by *KRAS/BRAF* wild-type tumors as a whole group with those collectively obtained from the group of tumors harboring such mutations, ERBB2 and ERBB4 were among the prevailing substrates discriminating these two groups. However, bearing in mind that the 11 discriminating substrates in this analysis appeared from a total number of 102 peptides constituting the tyrosine kinase activity profiles, the false discovery rate might be as high as 50% with the statistical significance level of *P*<0.05. Nevertheless, resistance to anti-EGFR antibody treatment may be mediated by activation of ERBB2-mediated signaling, either via amplification of *ERBB2* or increased levels of the ERBB3/ERBB4 ligand heregulin [13]. Moreover, *ERBB2* was recently found to be amplified in a third of tumors, predominantly colon cancer, confirmed to be wild-type for *KRAS/BRAF/PIK3CA* but resistant to anti-EGFR antibody therapy; tumors with *ERBB2* amplification were substantially enriched in this specific population compared to unselected patients [12]. In the present cohort of LARC patients, however, only two cases were concluded to have *ERBB2* amplification.

Specifically, using high-throughput kinase substrate arrays, an association between tumor kinase activity and metastasis-free survival was found in this LARC cohort. For clinical practice, this technology may be practicable, as it is robust with small tissue quantities, typically 10–15 micrograms of total protein being sufficient [4]–[6]; however, it has so far been employed to address a limited number of clinical topics [7], [8], [28]–[30]. The concept is contingent on fresh-frozen tumor tissue for preservation of kinase activity, and for the investigation reported here, we took the advantage of an existing biobank of biopsy samples prospectively compiled from study patients, enabling analysis of quality-assured tumor tissue. However, the present LARC population had not received anti-EGFR antibody treatment and thus, such outcome data was unavailable for correlation to the generated tumor kinase activity profiles.

In selecting cancer patients to kinase inhibiting therapeutics, the prevailing gold-standard is based mainly on detection of gene aberrations in the patients’ tumors. Such defects are embodied as the absence or presence of specific mutations, the latter being activating or inhibiting, or as amplifications or translocations, and are currently utilized in colorectal, breast, and non-small cell lung carcinomas, malignant melanoma, gastrointestinal stromal tumor, and some hematologic malignancies [19]. In the LARC population studied here, the observed frequencies of tumor aberrations of *KRAS*, *BRAF*, *PIK3CA*, and *ERBB2* were in the order of magnitude previously reported in colorectal cancer [9], [12], [17], [31]. Also in accordance with previous observations [9], mutations in *KRAS* and *BRAF* were mutually exclusive, whereas *KRAS* and *PIK3CA* mutations could coexist. The tumor from one patient harbored no less than four detected mutations (*KRAS* p.G12S, one *PIK3CA* exon 9 mutation, and two *PIK3CA* exon 20 mutations). Moreover, the lack of correlation between tumor *KRAS/BRAF* mutation status and treatment outcome for the present study population is in agreement with previous reports of other patient cohorts treated with neoadjuvant fluoropyrimidine−/oxaliplatin-based CRT [32]–[34]. Whether tumor *KRAS* mutation status is predictive for cetuximab-based CRT in LARC, is presently under debate [35]–[38].

In conclusion, recognizing that high tumor PI3K-mediated signaling activity was associated with poor metastasis-free survival in LARC, the strategy of exploring tumor kinase activities might be used to define functional biomarkers for risk assessment and treatment stratification. The present analysis needs to be repeated in more comprehensive patient populations, preferably with validated outcome data from adjuvant therapy, to ultimately prove diagnostic value for identification of patients with highly aggressive disease. Alternatively, as research tool, this approach for analyzing composite activities of signaling pathway effector proteins may be further developed to study actionable targets for prevention or treatment of colorectal cancer metastasis in general.

## Supporting Information

Table S1 **Specifications of the tumor** ***KRAS*** **,** ***BRAF*** **, and** ***PIK3CA*** **mutation analyses.**
(DOC)

Table S2 **The 102 array substrates generating the** ***ex vivo*** **tumor kinase activity signatures.**
(DOC)

Table S3 **Characteristics of 63 study patients with locally advanced rectal cancer with regard to tumor** ***KRAS/BRAF*** **mutation status.**
(DOC)
