# Supplementary material for: Tumor Phosphatidylinositol-3-Kinase Signaling and Development of Metastatic Disease in Locally Advanced Rectal Cancer
Source: PLoS One. 2012 Nov 30;7(11):e50806. doi: 10.1371/journal.pone.0050806 (PMC3511283; doi:10.1371/journal.pone.0050806)
Supplement: Table S1 — Specifications of the tumor KRAS , BRAF , and PIK3CA mutation analyses. (DOC) [file pone.0050806.s001.doc]

**Table S1.** Specifications of the tumor *KRAS*, *BRAF*, and *PIK3CA* mutation analyses.

| Primer | Sequence | Number of PCR^a^ cycles | PCR^a^ annealing temperature | CTCE^b^ separation temperature |
| --- | --- | --- | --- | --- |
| *KRAS* exon 2,  PCR 1 Forward | CTTAAGCGTCGATGGAGGAG | 35 | 58°C |  |
| *KRAS* exon 2, PCR 1 Reverse | AGAATGGTCCTGCACCAGTAA |
| *KRAS* exon 2, PCR 2 Forward | CGCCCGCCGCGCCCCGCGCC- CGTCCCGCCGCCCCCGCCCG- CCTCTATTGTTGGATCATATTC | 20 | 48°C | 51–48°C |
| *KRAS* exon 2, PCR 2 Reverse | ATGACTGAATATAAACTTGT |
| *BRAF* exon 15, Forward | CCCGCCGCCCCCGCCCGTCC- AGACAACTGTTCAA | 35 | 55°C | 47–44°C |
| *BRAF* exon 15, Reverse | TTCCTTTACTTACTACACCTC |
| *PIK3CA* exon 9, PCR 1 Forward | CATGCTGAGATCAGCCAAAT | 35 | 47°C |  |
| *PIK3CA* exon 9, PCR 1 Reverse | CGCCCGCCGCGCCCCGCGTG- GTTCTTTCCTGTCTCTGAAA |
| *PIK3CA* exon 9, PCR 2 Forward | CCCGCCGCCCCCGCCCGGAC- AAAGAACAGCTCAAAGCAA | 25 | 52°C | 46–43°C |
| *PIK3CA* exon 9, PCR 2 Reverse | GCTGAGATCAGCCAAATTCA |
| *PIK3CA* exon 20, Fragment 1 Forward | TATTCGACAGCATGCCAATC | 35 | 55°C | 49–46°C |
| *PIK3CA* exon 20, Fragment 1 Reverse | CCCGCCGCCCCCGCCCGTCC- AAAGCCTCTTGCTCAGT |
| *PIK3CA* exon 20, Fragment 2 Forward | ACATTCGAAAGACCCTAGCC | 35 | 55°C | 51–48°C |
| *PIK3CA* exon 20, Fragment 2 Reverse | CCCGCCGCCCCCGCCCGTGT- GTGGAAGATCCAATCCA |
| GC clamp^c^ | CGCCCGCCGCGCCCCGCGCC- CGTCCCGCCGCCCCCGCCCG |  |  |  |

^a^ Polymerase chain reaction

^b^ Cycling temperature capillary electrophoresis

^c^ Continuous stretch of G and C bases.
