# Supplementary material for: Tumor Phosphatidylinositol-3-Kinase Signaling and Development of Metastatic Disease in Locally Advanced Rectal Cancer
Source: PLoS One. 2012 Nov 30;7(11):e50806. doi: 10.1371/journal.pone.0050806 (PMC3511283; doi:10.1371/journal.pone.0050806)
Supplement: Table S2 — The 102 array substrates generating the ex vivo tumor kinase activity signatures. (DOC) [file pone.0050806.s002.doc]

**Table S2.** The 102 array substrates generating the *ex vivo* tumor kinase activity signatures.

| Substrate identity^a^ | Peptide sequence | Tyrosine position^b^ | Common name^a^ |
| --- | --- | --- | --- |
| ANXA1 | IENEEQEYVQTVK | [21] | Annexin A1 |
| ANXA2 | HSTPPSAYGSVKA | [24] | Annexin A2 |
| *ART-004* | EAIYAAPFAKKK | [4] | *Artificial peptide sequence* |
| BCKDHA | DDSSAYRSVDEVN | [345] | 2-oxoisovalerate dehydrogenase alpha subunit, mitochondrial |
| C1R | TEASGYISSLEYP | [204, 210] | Complement C1r subcomponent |
| CALM1 | KDGNGYISAAELR | [100] | Calmodulin |
| CBL | EGEEDTEYMTPSS | [700] | CBL E3 ubiquitin protein ligase |
| CD247 | KDKMAEAYSEIGM | [123] | T-cell surface glycoprotein CD3 zeta chain |
| CDK2 | EKIGEGTYGVVYK | [15, 19] | Cell division protein kinase 2 |
| CDK7 | GLAKSFGSPNRAY | [169] | Cell division protein kinase 7 |
| CHRND | YISKAEEYFLLKS | [383, 390] | Acetylcholine receptor protein, delta subunit |
| CTNNB1 | VADIDGQYAMTRA | [86] | Beta-catenin |
| CTTN1 | VSQREAEYEPETV | [477] | Src substrate protein p85 |
| CTTN1 | EYEPETVYEVAGA | [477, 483] | Src substrate protein p85 |
| CTTN1 | YQAEENTYDEYEN | [492, 499, 502] | Src substrate protein p85 |
| DCX | GIVYAVSSDRFRS | [112] | Neuronal migration protein doublecortin |
| DDR1 | LLLSNPAYRLLLA | [513] | Epithelial discoidin domain receptor 1 |
| DYRK1A | CQLGQRIYQYIQS | [319, 321] | Dual-specificity tyrosine phosphorylation regulated kinase 1A |
| EGFR | GSVQNPVYHNQPL | [1110] | Epidermal growth factor receptor |
| EGFR | ISLDNPDYQQDFF | [1172] | Epidermal growth factor receptor |
| EGFR | STAENAEYLRVAP | [1197] | Epidermal growth factor receptor |
| ENPEP | EREGSKRYCIQTK | [12] | Glutamyl aminopeptidase |
| EPB41 | LDGENIYIRHSNL | [660] | Protein 4.1 |
| EPHA1 | LDDFDGTYETQGG | [781] | Ephrin type-A receptor 1 |
| EPHA2 | EDDPEATYTTSGG | [772] | Ephrin type-A receptor 2 |
| EPHA4 | LNQGVRTYVDPFT | [596] | Ephrin type-A receptor 4 |
| EPHA7 | TYIDPETYEDPNR | [608, 614] | Ephrin type-A receptor 7 |
| EPHB1 | DDTSDPTYTSSLG | [778] | Ephrin type-B receptor 1 |
| EPHB4 | IGHGTKVYIDPFT | [590] | Ephrin type-B receptor 4 |
| EPOR | SEHAQDTYLVLDK | [368] | Erythropoietin receptor |
| EPOR | ASAASFEYTILDP | [426] | Erythropoietin receptor |
| ERBB2 | LDIDETEYHADGG | [877] | Receptor tyrosine-protein kinase erbB-2 |
| ERBB2 | PTAENPEYLGLDV | [1248] | Receptor tyrosine-protein kinase erbB-2 |
| ERBB4 | IVAENPEYLSEFS | [1284] | Receptor tyrosine-protein kinase erbB-4 |
| FER | RQEDGGVYSSSGL | [714] | Proto-oncogene tyrosine-protein kinase FER |
| FES | REEADGVYAASGG | [713] | Proto-oncogene tyrosine-protein kinase Fes/Fps |
| FGFR1 | TSNQEYLDLSMPL | [766] | Basic fibroblast growth factor receptor 1 |
| FGFR2 | TLTTNEEYLDLSQ | [769] | Fibroblast growth factor receptor 2 |
| FGFR3 | TVTSTDEYLDLSA | [760] | Fibroblast growth factor receptor 3 |
| FRK | KVDNEDIYESRHE | [387] | Tyrosine-protein kinase FRK |
| INSR | YASSNPEYLSASD | [992, 999] | Insulin receptor |
| INSR | SLGFKRSYEEHIP | [1355] | Insulin receptor |
| JAK1 | AIETDKEYYTVKD | [1022, 1023] | Tyrosine-protein kinase JAK1 |
| JAK2 | VRREVGDYGQLHETE | [570] | Tyrosine-protein kinase JAK2 |
| KRT6E | GAGFGSRSLYGLG | [62] | Keratin, type II cytoskeletal 6E |
| LAT | MESIDDYVNVPES | [200] | Linker for activation of T cells |
| LAT | EEGAPDYENLQEL | [255] | Linker for activation of T cells |
| LCK | RLIEDNEYTAREG | [394] | Proto-oncogene tyrosine-protein kinase LCK |
| MAPK1 | HTGFLTEYVATRW | [187] | Mitogen-activated protein kinase 1 |
| MAPK7 | AEHQYFMTEYVAT | [215, 220] | Mitogen-activated protein kinase 7 |
| MAPK10 | TSFMMTPYVVTRY | [223] | Mitogen-activated protein kinase 10 |
| MAPK12 | ADSEMTGYVVTRW | [185] | Mitogen-activated protein kinase 12 |
| MBP | ARTAHYGSLPQKS | [203] | Myelin basic protein |
| MBP | FGYGGRASDYKSA | [261, 268] | Myelin basic protein |
| MBP | GRASDYKSAHKGF | [268] | Myelin basic protein |
| MET | RDMYDKEYYSVHN | [1230, 1234, 1235] | Hepatocyte growth factor receptor |
| MST1R | SALLGDHYVQLPA | [1353] | Macrophage-stimulating protein receptor |
| MST1R | YVQLPATYMNLGP | [1353, 1360] | Macrophage-stimulating protein receptor |
| NCF1 | QRSRKRLSQDAYR | [324] | Neutrophil cytosol factor 1 |
| NTRK1 | HIIENPQYFSDAC | [496] | High affinity nerve growth factor receptor |
| NTRK2 | GMSRDVYSTDYYR | [702, 706, 707] | BDNF/NT-3 growth factors receptor |
| PDGFRB | VSSDGHEYIYVDP | [579, 581] | Beta platelet-derived growth factor receptor |
| PDGFRB | RPPSAELYSNALP | [716] | Beta platelet-derived growth factor receptor |
| PDGFRB | SSNYMAPYDNYVP | [771, 775, 778] | Beta platelet-derived growth factor receptor |
| PDGFRB | YMAPYDNYVPSAP | [771, 775, 778] | Beta platelet-derived growth factor receptor |
| PDGFRB | LDTSSVLYTAVQP | [1009] | Beta platelet-derived growth factor receptor |
| PDGFRB | PNEGDNDYIIPLPDP | [1021] | Beta platelet-derived growth factor receptor |
| PDPK1 | ARTTSQLYDAVPI | [9] | 3-phosphoinositide-dependent protein kinase 1 |
| PDPK1 | DEDCYGNYDNLLS | [373, 376] | 3-phosphoinositide-dependent protein kinase 1 |
| PECAM1 | KKDTETVYSEVRK | [713] | Platelet endothelial cell adhesion molecule |
| PGR | EQRMKESSFYSLC | [795] | Progesterone receptor (PR) |
| PIK3R1 | NENTEDQYSLVED | [607] | Phosphatidylinositol-3-kinase regulatory alpha subunit |
| PLCG1 | IGTAEPDYGALYE | [771, 775] | 1-phosphatidylinositol-4,5 bisphosphate phosphodiesterase gamma 1 |
| PPP2CB | EPHVTRRTPDYFL | [307] | Serine/threonine protein phosphatase 2A, catalytic subunit, beta isoform |
| PRRX2 | WTASSPYSTVPPY | [208, 214] | Paired mesoderm homeobox protein 2 |
| PTK2 | RYMEDSTYYKASK | [570, 576, 577] | Focal adhesion kinase 1 |
| PTK2B | RYIEDEDYYKASV | [573, 579, 580] | Protein tyrosine kinase 2 beta |
| PTPN11 | SKRKGHEYTNIKY | [546, 551] | Tyrosine-protein phosphatase, non-receptor type 11 |
| PXN | FLSEETPYSYPTG | [31, 33] | Paxillin |
| PXN | VGEEEHVYSFPNK | [118] | Paxillin |
| RAF1 | PRGQRDSSYYWEI | [340, 341] | RAF proto-oncogene serine/threonine protein kinase |
| RASA1 | TVDGKEIYNTIRR | [460] | Ras GTPase-activating protein 1 |
| RB1 | IYISPLKSPYKIS | [805, 813] | Retinoblastoma-associated protein |
| RET | TPSDSLIYDDGLS | [1029] | Proto-oncogene tyrosine-protein kinase receptor ret |
| SLC34A1 | AKALGKRTAKYRW | [511] | Renal sodium-dependent phosphate transport protein 2 |
| SYK | ALRADENYYKAQT | [525, 526] | Spleen tyrosine kinase |
| TEC | RYFLDDQYTSSSG | [513, 519] | Tyrosine-protein kinase Tec |
| TNNT1 | SDTEEQEYEEEQP | [9] | Slow skeletal muscle troponinT |
| TYRO3 | KIYSGDYYRQGCA | [681, 685, 686] | Tyrosine-protein kinase receptor TYRO3 |
| VCL | KSFLDSGYRILGA | [822] | Vinculin |
| VEGFR1 (FLT1) | DFGLARDIYKNPD | [1048] | Vascular endothelial growth factor receptor 1 |
| VEGFR1 (FLT1) | KNPDYVRKGDTRL | [1053] | Vascular endothelial growth factor receptor 1 |
| VEGFR1 (FLT1) | ATSMFDDYQGDSS | [1242] | Vascular endothelial growth factor receptor 1 |
| VEGFR1 (FLT1) | DYNSVVLYSTPPI | [1327, 1333] | Vascular endothelial growth factor receptor 1 |
| VEGFR2 (KDR) | RFRQGKDYVGAIP | [951] | Vascular endothelial growth factor receptor 2 |
| VEGFR2 (KDR) | EEAPEDLYKDFLT | [996] | Vascular endothelial growth factor receptor 2 |
| VEGFR2 (KDR) | DIYKDPDYVRKGD | [1054, 1059] | Vascular endothelial growth factor receptor 2 |
| VEGFR2 (KDR) | DFGLARDIYKDPD | [1063] | Vascular endothelial growth factor receptor 2 |
| VEGFR2 (KDR) | AQQDGKDYIVLPI | [1175] | Vascular endothelial growth factor receptor 2 |
| VEGFR3 (FLT4) | DIYKDPDYVRKGS | [1063, 1068] | Vascular endothelial growth factor receptor 3 |
| ZAP70 | ALGADDSYYTARS | [492, 493] | Tyrosine-protein kinase ZAP-70 |
| ZBTB16 | LRTHNGASPYQCT | [630] | Zinc finger and BTB domain containing protein 16 |

Single peptide substrates with significantly higher phosphorylation levels (*P* < 0.05) in the whole group of *KRAS/BRAF* wild-type tumors as compared with the group of *KRAS/BRAF* mutated tumors are shaded.

^a^ Retrieved from UniProtKB/SwissProt (<http://au.expasy.org/sprot>).

^b^ Position(s) of the tyrosine phosphorylation site(s) within the protein.
