# Supplementary material for: Tumor Phosphatidylinositol-3-Kinase Signaling and Development of Metastatic Disease in Locally Advanced Rectal Cancer
Source: PLoS One. 2012 Nov 30;7(11):e50806. doi: 10.1371/journal.pone.0050806 (PMC3511283; doi:10.1371/journal.pone.0050806)
Supplement: Table S3 — Characteristics of 63 study patients with locally advanced rectal cancer with regard to tumor KRAS/BRAF mutation status. (DOC) [file pone.0050806.s003.doc]

**Table S3.** Characteristics of 63 study patients with locally advanced rectal cancer with regard to tumor *KRAS/BRAF* mutation status.

|  |  | All patients (*n* = 63) | Patients with wild-type *KRAS/BRAF* tumor (*n* = 37) | Patients with mutated *KRAS/BRAF* tumor (*n* = 26) |
| --- | --- | --- | --- | --- |
|  |  | *n* (%) | *n* (%) | *n* (%) |
| TNM |  |  |  |  |
|  | T2 | 4 (6.3%) | 3 (8.1%) | 1 (3.8%) |
|  | T3 | 36 (57%) | 21 (57%) | 15 (58%) |
|  | T4 | 23 (37%) | 13 (35%) | 10 (38%) |
|  | N0 | 8 (13%) | 5 (14%) | 3 (12%) |
|  | N1 | 9 (14%) | 3 (8.1%) | 6 (23%) |
|  | N2 | 46 (73%) | 29 (78%) | 17 (65%) |
|  | M0 | 58 (92%) | 34 (92%) | 24 (92%) |
|  | M1 | 5 (7.9%) | 3 (8.1%) | 2 (7.7%) |
| ypTN | | |  |  |
|  | ypT0 | 13 (21%) | 8 (22%) | 5 (19%) |
|  | ypT1 | 6 (9.5%) | 3 (8.1%) | 3 (12%) |
|  | ypT2 | 16 (25%) | 11 (30%) | 5 (19%) |
|  | ypT3 | 16 (25%) | 8 (22%) | 8 (31%) |
|  | ypT4 | 12 (19%) | 7 (19%) | 5 (19%) |
|  | ypN0 | 48 (76%) | 29 (78%) | 19 (73%) |
|  | ypN1 | 11 (17%) | 6 (16%) | 5 (19%) |
|  | ypN2 | 4 (6.3%) | 2 (5.4%) | 2 (7.7%) |
| TRG^a^ |  |  |  |  |
|  | 1–2 | 45 (71%) | 26 (70%) | 19 (73%) |
|  | 3 | 10 (16%) | 5 (14%) | 5 (19%) |
|  | 4–5 | 8 (13%) | 6 (16%) | 2 (7.7%) |
| Metastatic disease^b^ |  | 20 (31.7%) | 11 (29.7%) | 9 (34.6%) |
| Median age (range), years | | 61 (31–73) | 61 (31–73) | 61 (42–72) |
| Gender |  |  |  |  |
|  | Male | 39 (62%) | 24 (65%) | 15 (58%) |
|  | Female | 24 (38%) | 13 (35%) | 11 (42%) |

^a^ Tumor Regression Grade following chemoradiotherapy.

^b^ Censored at a median period of 53 months (range 7–70).
